# Monitoring Inflammatory Markers and Anti-α-Gal Antibodies in Liver Transplant Recipients: Implications for Infection and Rejection

**DOI:** 10.3390/diagnostics16111635

**Published:** 2026-05-27

**Authors:** Gamze Karaca, Zeynal Mete Karaca, Basak Kayhan, Veysel Ersan, Baris Otlu, Elif Yesilada, Sezai Yilmaz

**Affiliations:** 1 Department of Medical Biology, Faculty of Medicine, Kırklareli University, Kırklareli 39100, Türkiye; 2 Department of Medical Genetics, Faculty of Medicine, Kırklareli University, Kırklareli 39100, Türkiye; zeynalmetekaraca@klu.edu.tr; 3 Department of Medical Genetics, Faculty of Medicine, İnönü University, Malatya 44280, Türkiye; elif.yesilada@inonu.edu.tr; 4 Department of Pharmaceutical Microbiology, Faculty of Pharmacy, Anadolu University, Eskişehir 26470, Türkiye; basakkayhan@anadolu.edu.tr; 5 Transplantation Immunology Laboratory, Liver Transplantation Institute, İnönü University, Malatya 44280, Türkiye; 6 Department of General Surgery, Liver Transplantation Institute, İnönü University, Malatya 44280, Türkiye; veysel.ersan@inonu.edu.tr (V.E.); sezai.yilmaz@inonu.edu.tr (S.Y.); 7 Department of Medical Microbiology, Faculty of Medicine, İnönü University, Malatya 44280, Türkiye; baris.otlu@inonu.edu.tr

**Keywords:** liver transplantation, alpha-Gal1,3, graft rejection, infection, immune response, gene expression

## Abstract

**Objective:** Living donor liver transplantation is a multifactorial process, and non-invasive serological parameters that may provide information about graft and patient status are still under investigation. However, time-dependent factors such as infection and rejection are often overlooked. Therefore, this study aimed to investigate anti-α-Gal1,3 levels and the expression of inflammatory and anti-inflammatory genes in peripheral blood samples obtained from 26 liver transplant patients before transplantation and at the first and sixth months post-transplantation. Additionally, 15 healthy volunteers were included as a control group, and patients were followed for two years to evaluate graft rejection. **Method:** A total of 26 living-donor liver transplant recipients and 15 healthy volunteers were included in the study. Peripheral blood samples were collected from patients before transplantation and at the first and sixth months after transplantation. Gene expression levels of *IL2*, *IL4*, *IL6*, *IL10*, *TNF*, *IFNG*, *FOXP3*, *TREM1*, *CD14*, and *HLAG5* were analyzed by qRT-PCR, while anti-α-Gal IgM and IgG levels were measured by ELISA. In addition, biochemical parameters and microbiological culture analyses were evaluated, and bacterial identification was confirmed by MALDI-TOF MS when necessary. Analyses were performed across three time points with respect to infection status and graft rejection. **Results:** While the expression of *IFNG*, *IL2*, and *HLAG5* significantly increased 6 months after transplantation, *IL10* expression decreased significantly. In patients without rejection, IFNG expression increased significantly and IL10 expression decreased significantly at the sixth month. Microbiological evaluation showed that infections were more frequent, particularly during the first three months after transplantation. While anti-α-Gal IgG levels did not differ according to infection status, IgM levels decreased significantly at the sixth month, and this decline was associated with a reduced infection burden. **Conclusions:** In conclusion, *IFNG* and *IL10* gene expression levels may serve as important indicators for predicting rejection after liver transplantation, whereas anti-α-Gal IgM levels may provide useful guidance in monitoring infection.

## 1. Introduction

Liver transplantation (LT) is the most effective treatment for advanced chronic liver failure caused by various diseases. It not only prolongs life expectancy, which is often limited to days or months before transplantation, but also significantly improves the quality of life [1,2,3].

Advances in immunosuppressive treatments and surgical techniques have notably increased graft survival rates after LT. Despite these improvements, postoperative complications remain a significant challenge. Immunological factors and postoperative infections are among the leading causes of these complications [4,5]. Therefore, monitoring the immune system and infection status of a recipient’s post-transplantation is crucial. However, standard analyses may not always yield consistent results due to variability in the recipient’s underlying medical condition, age, metabolic problems, infectious factors, and the mechanisms of action of immunosuppressive drugs [6,7,8].

In case of infection, the intensity and timing of infection exposure, as well as the virulence of the pathogen, play a pivotal role in morbidity and mortality after LT. Bacterial infection patterns vary over time following transplantation [6,9,10]. Nosocomial organisms and the recipient’s normal flora are the primary causes of infections during the first month post-transplantation, although donor-derived infections can also occur. From the second to sixth months, opportunistic infections become more prominent due to patient risk factors and the intensity of immunosuppressive therapy [6,10,11,12].

These infections highlight the need for faster, more sensitive, and cost-effective alternatives to traditional microbiological and biochemical analyses. Galactose-alpha-1,3-galactose (α-Gal) is an epitope abundantly expressed on glycolipids and glycoproteins of non-primate mammals and new-world monkeys. In humans, the enzyme alpha-1,3-galactosyltransferase, which synthesizes this epitope, is lost evolutionarily [13]. Consequently, the human immune system produces anti-α-Gal antibodies, constituting approximately 1% of serum immunoglobulins and serving as the most abundant natural antibody [14]. Bacteria in the intestinal microbiota are shown to be responsible for this since anti-α-Gal binds to various bacteria and bacterial lipopolysaccharides. Although the exact bacterial carbohydrates inducing anti-α-Gal production remain unidentified, Galα1-3Glc and Galα1-3Gal epitopes have been reported in both gram-positive and gram-negative bacteria [15,16]. Anti-α-Gal antibodies can also bind to protozoa, enveloped viruses, and some pathogenic bacteria, making them potential candidates for early detection of post-LT infections [14].

Infections caused by intracellular and extracellular pathogens activate different immune pathways involving cellular and soluble factors. Contact between antigen-presenting cells and antigens in the liver triggers a cellular immune response. Graft rejection is linked to the preferential activation of Th1-type CD4+ T-lymphocytes, which predominantly produce IL-2, IL-12, and IFN-γ. In contrast, prolonged allograft survival correlates with increased *IL4*, *IL10*, and *IL13* expression, driven by Th2 and regulatory CD4^+^ T cells [17]. Investigating soluble factors, cellular components, and transcription factors, in addition to inflammatory and anti-inflammatory cytokines, is essential for a comprehensive understanding of immune regulation. The *FOXP3* and its protein product play a crucial role in the development and function of Tregs, regulating T-lymphocyte activation and suppressing inflammatory cytokine production [18]. Studies indicate that decreased *FOXP3* expression within the first week after LT may be associated with acute rejection. TREM-1 and HLA-G5 are receptors with opposing immunomodulatory roles. TREM-1 amplifies the pro-inflammatory cytokine response upon bacterial and fungal infections, whereas HLA-G5 exerts its immunosuppressive effects through both innate and adaptive immune pathways. Elevated HLA-G5 levels in transplant recipients have been correlated with enhanced graft tolerance and acceptance [19,20,21].

This study aims to provide a comprehensive assessment of immune response and infection status in LT recipients by analyzing the expression levels of inflammatory (*IL2*, *IL6*, *TNF*, *IFNG*, *TREM1*, *CD14*) and anti-inflammatory (*IL4*, *IL10*, *FOXP3*, *HLAG5*) genes at three time points before and after transplantation. These findings are correlated with biochemical parameters, microbiological cultures, and anti-α-Gal IgM and IgG levels to offer valuable insights into post-LT immune regulation and infection monitoring, as well as a two-year follow-up in case of rejection.

## 2. Materials and Methods

### 2.1. Study Population and Immunosuppression Protocol

The study was approved by the local ethics committee (Protocol No: 2020/24, Date: 5 February 2020) and was conducted in accordance with the ethical standards of the Declaration of Helsinki and its later amendments. The study included 26 patients (10 women and 16 men) scheduled for liver transplantation due to end-stage liver disease of different etiologies. The control group consisted of 15 healthy volunteers (7 women and 8 men) without any known disease. Individuals with a history of trauma, autoimmune disease, HIV infection, pregnancy, acute or chronic pancreatitis, hepatitis B or C infection, burns, chronic renal failure, steroid use, neutropenia, diabetes, malignancy, or immunosuppressive drug use were excluded from the control group. The purpose of the study and the tests to be conducted were explained to all participants, and informed consent was obtained. Participants were between 18 and 65 years old, and all transplants were performed using living donors. The clinical characteristics of the patients and the distribution of diseases leading to transplantation are summarized in Table 1. Measurements obtained from healthy volunteers are denoted as “Control.” Patient group measurements at different time points are categorized as follows: “Preop” refers to preoperative measurements, “Postop I” refers to measurements taken one month postoperatively, and “Postop VI” refers to measurements taken six months postoperatively. The diagnosis of rejection was made based on serological assessment, radiological evaluation of graft function, and the Banff criteria.

According to the protocol of our center, immunosuppressive therapy typically begins with intravenous steroid administration following graft anastomosis. In the postoperative period, oral steroid treatment was started at 80 mg/day and gradually tapered daily. Tacrolimus was started at a dose of 0.02 mg/kg/dose if renal function tests were normal. If tacrolimus induced renal dysfunction, its dose was reduced or discontinued, and everolimus was introduced [22]. The use of tacrolimus and everolimus over the six-month postoperative period varied depending on renal function status. The details of tacrolimus and everolimus usage are provided in Table 2.

### 2.2. Biochemical and Microbiological Analysis Methods

After the surgery, all patients were taken to the intensive care unit (ICU); blood and urine cultures of all liver transplant patients were routinely obtained upon admission to the ICU for biochemical and microbiological analysis.

All biochemical parameters related with liver function [aspartate aminotransferase (AST), alanine transaminase (ALT), alpha fetoprotein (AFP), C-reactive protein (CRP), direct bilirubin (Bilirubin D), total bilirubin (Bilirubin T)] in serum samples were studied in an automated system (Abbott Architect C8000, Abbott Laboratories, Abbott, IL, USA) with quantitative tests. Thrombocyte levels (PLT) were studied in a Sysmex XN-1000 hematology analyzer (Sysmex Corporation, Kobe, Japan).

Microbiological analyses of blood samples were incubated in the BACT/ALERT 3D (BioMérieux, Marcy-l’Étoile, France) automated blood culture system. Positive samples were plated on 5% sheep blood agar, Eosin Methylene Blue (EMB) agar, and chocolate agar and incubated at 35–37 °C for 18–24 h. Growing bacterial colonies were selected and identified by classical bacteriological analyses for other samples. In patients where classical methods were insufficient and species identification was required, bacterial colonies were identified by “Matrix-Assisted Laser Desorption/Ionization Time-of-Flight Mass Spectrometry” (MALDI-TOF MS) (BioMerieux, Marcy-l’Étoile, France).

### 2.3. Real-Time Polymerase Chain Reaction

Peripheral blood samples were taken from the healthy control group and patients immediately before surgery, and 1 and 6 months after surgery for analysis of gene expressions. Total RNA samples were extracted by using a QIAamp RNA Blood Mini Kit (Qiagen, Hilden, Germany) according to the manufacturer’s instructions. The quality and quantity of RNA samples were evaluated by a MaestroNano Spectrophotometer (Maestrogen Inc., Taiwan). RNA samples with an A260/280 ratio of approximately 2.0–2.3 were used in cDNA synthesis. The RNA purification process of samples with inappropriate absorbance values was repeated. RNA samples were visualized using 1% agarose gel before and after heating the samples at 70 °C to eliminate the artificial genomic structure.

One microgram of RNA was used for cDNA synthesis, and reverse transcription was performed by using an RT^2^ HT First Standard Kit (Qiagen) according to the manufacturer’s protocol. For RT^2^-qPCR reactions, cDNA was mixed with SYBR^®^ Green qPCR FAST Mastermix (RT^2^ SYBR^®^ Green FAST Mastermixes; Qiagen) and then aliquoted into the tubes containing commercially provided primers for *IL2*, *IL4*, *IL6*, *IL10*, *TNF*, *IFNG*, *FOXP3*, *TREM1*, *CD14*, *HLA-G5*, and housekeeping gene *GAPDH* (Qiagen). Features of all these primers are presented in Table 3. Reactions were performed at a final volume of 25 mL containing 12.5 mL of RT^2^ SYBR Green Mastermix, 6.5 mL of RNAase free water, 5 mL of cDNA, and 1 mL of RT2-qPCR primers. PCR conditions were performed according to the manufacturer’s instructions and repeated for 40 cycles. The array was run on a QIAGEN-Rotor Gene Q (Qiagen), and data were analyzed by using the 2^−ΔCycle^ threshold (Ct) method. Samples were normalized to housekeeping gene *GAPDH* by using the RT^2^ Profiler PCR Array Data Analysis version 3.5 software analysis program.

PCR products were visualized on a 2% agarose gel. A 100 bp standard DNA sample (GelPilot 100bp DNA Ladder, Qiagen) and 10 μL of PCR product with 2 μL of 6× loading dye for each gene were mixed and run on a 2% agarose gel for 30 min at 100 volts. Gel images were obtained and recorded on a gel imaging device (KODAK Gel Logic 2200 Imaging System, Carestream Health, Rochester, NY, USA).

### 2.4. Enzyme-Linked Immunosorbent Assay (ELISA)

Anti-α-Gal serum concentrations were measured by using human anti-α-galactosyl IgG (Cat. No. RD199178100R) and IgM (Cat. No: RD199178110R) ELISA kits according to the manufacturer’s instructions (BioVendor, Laboratorní Medicína a.s., Brno, Czech Republic). Optical density values were determined by using an ELISA reader at 450 nm reference filter (BİOTEK ELx800, Microplate Reader, BioTek Instruments, Winooski, VT, USA). After the measurement, anti-α-Gal IgG and IgM levels in the samples were determined using the optical density values of the standards at known concentrations in the kit. The detection range for both kits is stated as 3.3–100 U/mL.

### 2.5. Statistical Analysis

Data were analyzed using SPSS 25.0 (Statistical Package for the Social Sciences, Inc., Chicago, IL, USA). Normality of data distribution was assessed using the Shapiro–Wilk test. As the patient group did not exhibit a normal distribution in repeated pre- and post-transplant measurements, the Friedman Test was used to evaluate differences over time. The Wilcoxon Test was performed to identify specific differences between time points following a significant Friedman Test result, with Bonferroni correction applied to account for potential alpha errors. Pairwise comparisons between the control and transplantation groups at different time points were conducted using the Mann–Whitney U test. To assess correlations between gene expression levels across different time points, Spearman correlation analysis was performed. For qualitative data, Cochran’s Q test was applied to evaluate statistical differences between periods. A *p*-value of less than 0.05 was considered statistically significant.

## 3. Results

Considering the risk of infection in the first six months after liver transplantation, analyzing gene expression patterns related to inflammatory and anti-inflammatory immune responses alongside infection-associated parameters at different time points provides valuable insights for patient follow-up. Therefore, in this study, biochemical, microbiological, and molecular immunology analyses were conducted using biological samples obtained from recipients before and after liver transplantation (at 1 and 6 months).

Among the 26 liver transplant recipients, 16 were male and 10 were female, with a median age of 55 years. The mean Model for End-Stage Liver Disease (MELD) score was 17.73 ± 4.98. The etiologies of these patients are summarized in Table 1.

### 3.1. Biochemical and Microbiological Analysis Results

The patients’ ALT, AST, AFP, PLT, CRP, and direct and total bilirubin levels were analyzed before the operation, and 1 and 6 months after the operation (Table 4). Among these parameters, ALT, PLT, and CRP values were found to increase significantly in the first month after the operation compared to the pre-operative period, while they decreased significantly 6 months after the operation compared to the first month. On the other hand, AST and bilirubin D values showed significant reduction in the first and sixth months after the operation compared to the preoperative measurement. Among the biochemical parameters, the AFP and bilirubin T values decreased significantly in the first and sixth months after transplantation compared to the pre-transplantation period.

According to the analysis of blood, sputum, urine, and wound samples, microorganisms were identified in 17 patients (Table 5). No signs of infection were detected in nine patients, and culture results taken for control purposes were negative. The number of identified microorganisms is significantly higher than in the first three months after liver transplantation in comparison to 3–6 months after the liver transplantation period (*p* < 0.05). Pathogen-specific descriptive anti-α-Gal IgM and IgG profiles are provided in Supplementary Table S1.

### 3.2. Gene Expression Analysis

**Figure 1:**
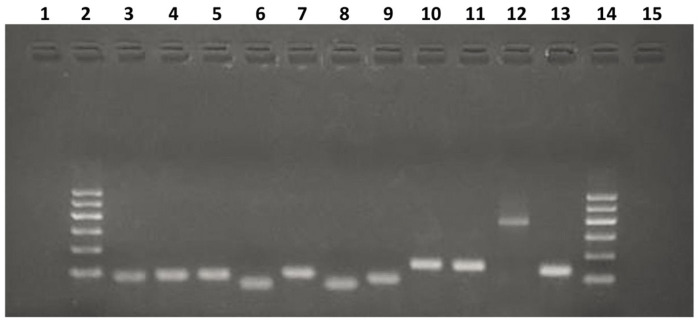
Representative image of PCR products. DNA samples after qRT-PCR and visualization of those PCR products at 2% gel electrophoresis (45 min, 90 V). Line 2 and line 14 represent 100 bp DNA marker (Gelpilot; QiaGen), 3rd–13th lines represent the qRT-PCR product for *IL-2* (85 bp), *IL-4* (93 bp), *IL-6* (98 bp), *IL-10* (63 bp), *TNF-a* (110 bp), *IFN-g* (68 bp), *Foxp3* (89 bp), *TREM-1* (156 bp), *CD14* (152 bp), *HLA-G5* (384 bp), and the house-keeping gene *GAPDH* (130 bp), respectively.

**Figure 2:**
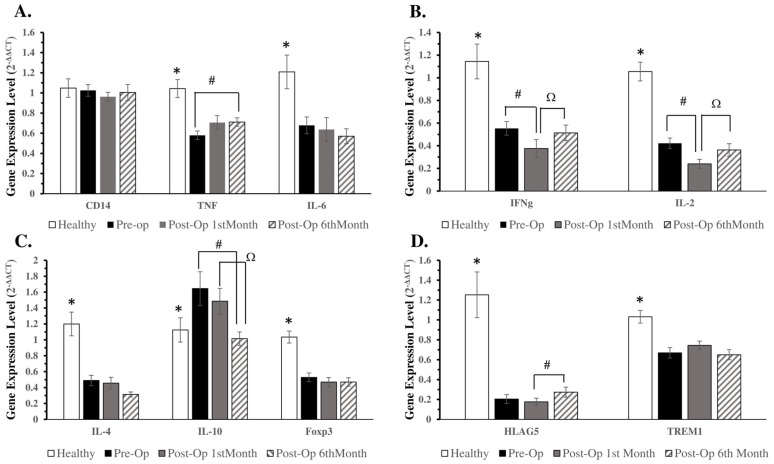
Gene expression levels. Gene expression levels of inflammatory (*IFN-g*, *TNF-a*, *IL-2*, *IL-6*, *TREM1*, *CD14*) and anti-inflammatory (*IL-10*, *IL-4*, *HLA-G5*, *foxp3*) factors. The data represent the mean ± SD of 2^(Avg−Δ(ΔCt))^ values in two separate experiments. Figure (**A**) shows the intergroup mean gene expression differences of *CD14*, *TNF-a*, and *IL-6* genes; Figure (**B**) shows *IFN-g* and *IL-2* genes; Figure (**C**) shows *IL-4, IL-10*, and *FOXP3* genes; and Figure (**D**) shows *HLA-G5* and *TREM1* genes. In the figure, * symbolizes that the values in the healthy control group increased (*Tnf*, *Il6*, *Ifng*, *Il2*, *Il4*, *foxp3*, *Hlag5*, and *Trem1*) and decreased (*Il10*) significantly in comparison to other groups. The symbols # and Ω represent significant differences between the compared groups as mentioned in the figure.

The expression of pro-inflammatory (IL-6, TNF-a), inflammatory (IFN-g, IL-2), and anti-inflammatory (IL-4, IL-10) cytokines and cellular factors (CD14, foxp3, HLA-G5, TREM-1) in peripheral blood of healthy volunteers and patients were analyzed. The accuracy of the DNA fragment amplified after qRT-PCR was assessed in gel electrophoresis (Figure 1). Gene expressions of healthy individuals were found to be significantly higher than the expressions of patients at all time points in all parameters except *CD14* and *IL10* (Figure 2; *p* < 0.05).

In comparison to the pre-transplant period, *TNF* expression in the sixth month after transplantation increased significantly (Figure 2A; *p* < 0.05). When Th1 cytokine gene expressions were analyzed in patients before and after transplantation, it was found that *IFNG* expression decreased significantly in the first month after transplantation compared to the pre-transplant period. On the other hand, it increased significantly in the sixth month after transplantation compared to the first month after transplantation (Figure 2B; *p* = 0.026 and *p* = 0.010, respectively). A similar pattern was also observed in *IL2* expression (Figure 2B; *p* = 0.001 and *p* = 0.041).

The expression analysis of *IL4*, *IL10*, *FOXP3*, and *HLAG5*—genes that are related to immunological tolerance—differ from inflammatory response genes analysis (Figure 2C,D). Among them, significant reduction was detected only in the expression of *IL10*. Interestingly, *IL10* expression levels significantly reduced 1.62 times 6 months after transplantation in comparison to the pre-op period (*p* = 0.008). Furthermore, *IL10* expression 6 months after operation was 1.46-fold lower than the 1-month post-op level (*p* = 0.012). *HLAG5* expression increased significantly by 1.55-fold at 6 months post-transplant in comparison to 1 month post-op (*p* = 0.007) (Figure 2D).

**Figure 3:**
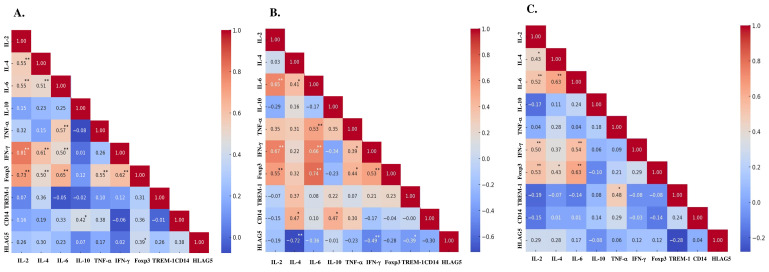
Correlation analysis. Correlation analyses of gene expression levels at different time periods. Figure (**A**) shows the analysis with correlation coefficient values for pre-op versus post-op 1 month. Figure (**B**) shows the analysis with correlation coefficient values for pre-op versus post-op 6 months, and figure (**C**) shows the analysis with correlation coefficient values for post-op 1 month versus post-op 6 months. The symbol ‘*’ indicates statistical significance at *p* < 0.05 level. The symbol ‘**’ indicates statistical significance at *p* < 0.01 level.

Correlation analyses of changes in gene expressions at different time periods were performed. In terms of positive correlation, when the pre-operative and first month postoperative data were compared, significant positive correlations were detected among *IL2*-*IFNG*, *IL2*-*FOXP3*, and *IL6*-*FOXP3* (correlation coefficient 0.81, 0.73, and 0.65, respectively) (Figure 3A). While the correlation between *IL2*-*IFNG* and *IL2*-*FOXP3* decreased, the correlation between *IL6*-*FOXP3* increased in pre-op versus sixth-month post-op time periods (correlation coefficient 0.67, 0.57 and 0.74, respectively) (Figure 3B). In the correlation analysis of data from the postoperative first month and the postoperative sixth month, it was determined that *IL6-IL4* and *IL6-FOXP3* showed a significant positive correlation (correlation coefficient 0.63 and 0.63, respectively) (Figure 3C). Interestingly, the analyses with the lowest coefficients in terms of negative correlation were found in the comparison of the pre-operative data and the sixth-month postoperative data. In that case, *IL4-HLAG5*, *IFNg-HLAG5*, and *TREM1-HLAG5* comparisons were the most prominent ones (correlation coefficient −0.72, −0.49, and −0.39, respectively) (Figure 3B).

### 3.3. Gene Expressions of Patients with and Without Rejection

Gene expression values of 8 transplant patients with rejection and 18 patients without rejection in the 2-year period after the operation were compared at the first and sixth months after the operation. Interestingly, it was found that *TNF* expression was significantly lower in patients who developed rejection in comparison to patients who did not experience rejection 1 month after transplantation. When we exclude rejection patients and compare gene expression analyses between patients without rejection at two different time points, *IL10* expression decreases significantly, while *IFNG* gene expression increases significantly at the end of 6 months in comparison to 1 month (*p* = 0.029 and *p* = 0.040, respectively) (Table 6).

### 3.4. Anti-α-Galactosyl IgM and IgG Levels Before and After Liver Transplantation

**Figure 4:**
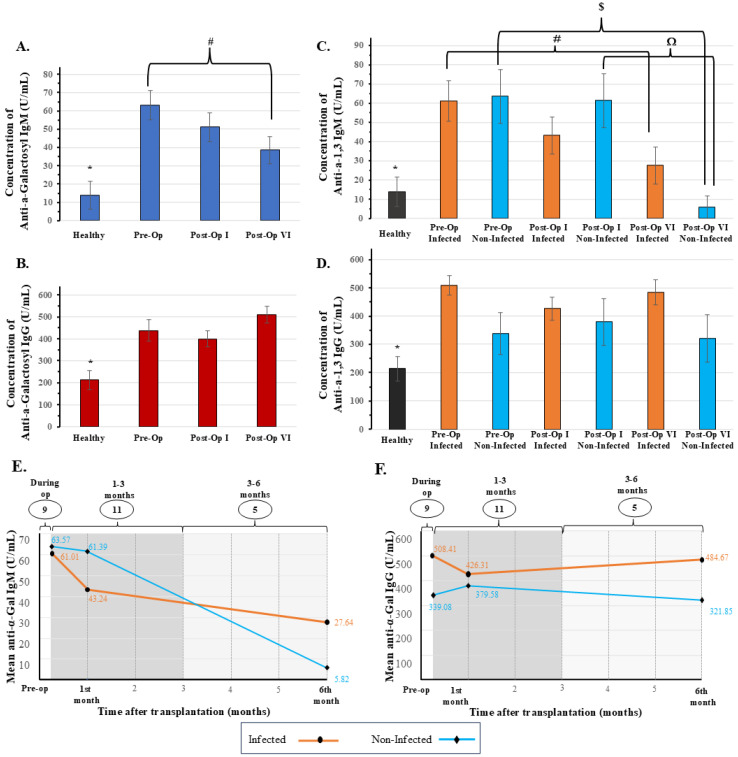
Anti-a-galactosyl IgM and IgG levels. Levels of anti-α-galactosyl IgM (**A**,**C**) and anti-α-galactosyl IgG (**B**,**D**) in serum samples of patients and healthy volunteers. Immunoglobulin levels of liver transplant patients were determined at three time points pre-op, post-op 1 month (Post-op I), and post-op 6 month (Post-Op VI), respectively. Anti-α-galactosyl IgM and IgG levels of liver transplant patients were also analyzed according to their infection status (**C**,**D**). Longitudinal line graphs show the mean anti-α-galactosyl IgM (**E**) and anti-α-galactosyl IgG (**F**) levels in infected and non-infected groups across the predefined sampling time points. Numbers in circles in panels (**E**,**F**) indicate culture-positive microorganism detections in each postoperative interval. The symbol ‘*’ indicates the statistical differences at IgM and IgG concentrations between the healthy group and liver transplant patient at three time points. The symbol ‘#’ indicates the statistical differences between pre-op and post-op 6 months (**A**) and differences between pre-op infected and 6-month post-op infected groups (**C**). The symbol ‘$’ indicates the statistical differences between pre-op non-infected and 6-month post-op non-infected groups (**C**). The symbol ‘Ω’ indicates the statistical difference between post-non infected I and post-op non-infected VI groups.

In terms of organ transplantation, alpha-Gal epitopes are one of the major risk epitopes encountered in xenotransplantation and have also been identified in the structures of many bacteria from a microbiological perspective. Therefore, anti-α-galactosyl levels in IgM and IgG isotypes are a matter of curiosity in both healthy individuals and recipients before and after transplantation (Figure 4). Both IgM and IgG levels were significantly increased in liver transplant recipients before and after transplantation compared to healthy individuals (Figure 4A,B) (*p* < 0.05 for both IgM and IgG). Furthermore, a significant difference was observed between IgM levels of the pre-op period and 6 months post-op (Figure 4A) (*p* = 0.001).

Anti-α-galactosyl IgM levels in all infected and non-infected groups, except for the uninfected group in the 6-month postoperative group, were found to be significantly higher than the healthy group (Figure 4C). In addition, it was determined that the IgM level of infected patients in the pre-operative period decreased significantly by approximately 2.21 times in the sixth month postoperatively (Figure 4C,E; *p* = 0.022). IgM levels of the pre-op non-infected and postoperative first month non-infected groups decrease by 10.92 times and 10.54 times, respectively, in the sixth postoperative month (Figure 4C,E; *p* = 0.017 and *p* = 0.028, respectively).

## 4. Discussion

Approximately 60% of all infections after liver transplantation are bacterial, 30% are viral, and 10% are fungal. The timing of infections following OLT is generally classified as early, intermediate, and late infections. These are classified as the first month after OLT, during months 1–6, and after 6 months, respectively. Infections that develop in the first month after transplantation are generally associated with surgery and are similar to infections seen in all patients who have undergone surgery [4]. Sources of infection are endogenous flora of the gastrointestinal tract and nosocomial agents in which predominantly bacterial and partly fungal infections are observed. Infections developing 1–6 months after OLT are at their highest level due to the suppression of the cellular immune response and therefore infectious agents are typically intracellular agents and opportunistic pathogens. The defined time intervals and frequency of infections may vary depending on the net immunosuppressive level of the patients and the developing comorbidities [8,9]. The ultimate goal of researchers is to discover specific parameters that can be used in the early diagnosis of infection and rejection after solid organ transplantation using non-invasive methods. However, before aiming for this goal, it may be an important approach to have knowledge about the kinetics of the target parameters in the pre- and post-transplant periods and during infection periods. In that study, the expression of genes that are effective in inflammatory and anti-inflammatory responses in recipients before and after transplantation, the source and agents of infections developing in patients, and the levels of IgM and IgG developed against the α-1,3 Gal molecule, which is also identified in many bacterial structures, were investigated.

IL-2 is a cytokine defined as an immunostimulatory factor that promotes the proliferation and differentiation of T cells. The aim of immunosuppressive therapy administered immediately before and/or after liver transplantation is to prevent the development of acute rejection caused by host T cells reacting predominantly against major and minor histocompatibility antigens. Calcineurin inhibitors (tacrolimus, cyclosporine) block IL-2 production and subsequently T cell proliferation. They stop T cell proliferation at a therapeutic concentration. Mammalian target of rapamycin (mTOR) inhibitors (sirolimus and everolimus) block the ability of IL-2 and IL-2 receptors to activate T cells through disruption of signaling pathways [23]. Zhang et al. stated in their study that by blocking the IL-2 receptor, the rate of exposure to acute rejection was lower compared to the control group. As a result of our research, it is thought that the decrease in IL-2 gene expression measured in the first month after the operation is due to the effect of the immunosuppressive protocol applied to the patient. The significant elevation on IL-2 expression at the sixth-month period may have been caused by abandoning tacrolimus due to its side effects on kidney functions and continuing the treatment with everolimus and reducing the dosage.

TNF-α works together with IL-6 to stimulate the cell cycle and initiate regeneration. This process involves all mature parenchymal and nonparenchymal cells, including hepatocytes, biliary epithelial cells, sinusoidal endothelial cells, Kupffer cells, and stellate cells. In particular, specific cytokines such as interleukin IL-6 and tumor necrosis factor (TNF)-α play an important role in the liver regeneration process [24,25]. Min Suk et al. reported an increase in serum TNF-α levels before surgery in their study of 226 liver transplantation patients. They associated higher preoperative serum TNF-α levels with liver graft regeneration after transplantation [26]. In our study, the stable level of IL-6 and the significant increase in TNF-α 6 months after LLT compared to the pre-transplant period may be associated with regeneration.

Our analyses show that IFN-γ acts in parallel with IL-2, and they are correlated with each other. This may be due to the effect of immunosuppressants; therefore, it would be appropriate to monitor IL-2 and IFN-γ throughout immunosuppressive therapy. Indeed, Millán et al. stated that IFN-γ should be monitored as a biomarker in transplantation to determine individual response to immunosuppressive therapy. They showed that effector T-cell response evaluated by intracellular expression of IFN-γ and IL-2 in CD4^+^ and CD8^+^-T cells and soluble synthesis of IFN-γ were increased in the rejection group in comparison to non-rejector ones [27]. In our results, the reason for the increase in IFN-g 6 months after transplantation may be due to the difference in immunosuppressive treatment options, and the infections that develop 1–6 months after the operation are likely to be caused by intracellular bacteria.

IL-10 is a key anti-inflammatory cytokine that acts as a negative regulator of the immune response to foreign antigens. It is known that genetic transfer of IL-10 into allotransplants leads to an increased graft acceptance rate in experimental heart or liver transplantation models. With its anti-inflammatory properties, IL-10 plays a critical role in limiting the immune response after infection but may also contribute to the triggering of chronic infection [28,29]. Various studies have shown that serum IL-10 levels can increase up to 50–60 times compared to preoperative values during living donor liver transplantation surgery or within the first hour of transplantation [30,31]. On the other hand, Karakhanova et al. concluded in their study on 41 liver transplant recipients that the IFN-γ level in the closest preoperative period may serve as a predictive parameter for early allograft dysfunction (EAD) and, in addition, the increase in IL-10 and CXCL10 (IP-10) levels in the early postoperative period may be prognostic for EAD [32]. Similarly, Kim et al.’s study on 52 liver transplant recipients showed that combined detection of IL-10, IL-17, and interferon gamma-inducible protein 10 (IP-10) in the first weeks after transplantation was a predictive marker of acute graft rejection with 94% sensitivity and 97% specificity [33]. As can be understood, although IL-10 is an active cytokine that plays a role in immune tolerance and can restrain the inflammatory response, increased expression during rejection may be explained by the activation of certain Treg subpopulations in response to inflammation and an attempt to maintain the IFN-γ/IL-10 balance in the allotransplant.

In solid organ transplantation, HLA-G interacts with CD4 T cells and dendritic cells involved in the initiation of the immune response. HLA-G promotes the Th2 response by suppressing Th1+ CD4 T cell proliferation in response to allogeneic stimulation. Therefore, it is stated that HLA-G has immunoregulatory properties like Tregs [34]. In a study conducted on 19 combined liver/kidney transplantation patients, Le Rond et al. showed that in patients with high HLA-G5 serum levels with no graft rejection, HLA-G5 inhibited T cell responses, that serum HLA-G5 was responsible for this inhibition, and that HLA-G5 promoted better survival [21]. Hu et al., investigated the expression of HLA-G in blood and liver tissue samples to determine the correlation between the expression of HLA-G and acute rejection in patients undergoing liver transplantation. In their research on 59 different tissue and blood samples, high expression of the HLA-G was shown to correlate with a reduced occurrence of acute liver rejection [35]. In our study, it was determined that the HLA-G5 gene expression 6 months after the operation showed a significant increase compared to the values 1 month later, when infectious agents were intense.

Human anti-α-Gal antibodies have been shown to interact with many GI bacteria, including *Escherichia coli*, *Klebsiella*, and *Salmonella* strains. The fact that postnatal anti-α-Gal antibodies are low in the first 6 months when the intestinal microflora is not yet formed and increase rapidly in the following period supports this hypothesis [36]. Jensen et al. showed that anti-α-Gal IgG antibodies isolated from samples collected from individuals diagnosed with sepsis could bind to 56 out of 100 pathogens that cause sepsis, including various pathogens such as *Enterococcus faecium*, *Staphylococcus aureus*, *Streptococcus pyogenes*, and *Escherichia coli*. They conclude that, although IgG anti-α-Gal comprises a small fraction of the human antibody pool (~0.1%), these antibodies target an impressively large part of pathogens causing invasive disease [37]. In our study, a consistent relationship was observed between the positivity of culture results obtained from liver transplant patients and the amount of anti-α-Gal IgM antibodies.

More specifically, anti-α-Gal IgM levels showed a significant postoperative decline over time. This decrease may be partly related to restoration of liver graft function after transplantation. In our cohort, the postoperative decrease in biochemical indicators of liver injury and cholestasis, including ALT, AST, and bilirubin, supported the improvement of graft function. The liver is known to play a central role in the clearance of circulating immune complexes through Kupffer cells and liver sinusoidal endothelial cells. In line with this concept, Lebherz–Eichinger et al. [38] reported that liver transplantation can reduce elevated circulating immunoglobulin levels in patients with chronic hepatic failure, which they attributed to reconstituted hepatic antibody clearance. Therefore, restoration of hepatic clearance capacity after transplantation may contribute to more efficient removal of circulating immune complexes and antigen–antibody structures, thereby reducing serum anti-α-Gal IgM levels. However, in patients with infectious complications, ongoing microbial antigen exposure and systemic inflammation may impose an additional burden on the graft and may alter or delay immune complex clearance. This may explain why anti-α-Gal IgM dynamics differed between infected and non-infected recipients. In this context, anti-α-Gal IgM dynamics may provide additional information, together with AST, ALT, and bilirubin, for monitoring immunological and functional recovery after liver transplantation.

In clinical practice, rejection is generally evaluated by serological/biochemical assessment, radiological evaluation of graft function, and histopathological assessment according to the Banff criteria [39,40], whereas the more challenging scenario is often the presence of infection-like findings such as fever, elevated CRP or procalcitonin, and increased liver enzymes despite negative or pending culture results [41]. In additional exploratory analyses, anti-α-Gal IgM levels were also compared between rejection and non-rejection groups; however, no statistically significant difference was observed, and therefore, these data were not presented as primary findings. Anti-α-Gal IgM showed a significant postoperative decline in both infected and non-infected recipients. The more pronounced decline observed in the non-infected group may be related to restoration of graft function and reconstituted hepatic immune-complex clearance, whereas ongoing microbial antigen exposure and systemic inflammation in infected recipients may alter this dynamic process. Therefore, anti-α-Gal IgM should not be interpreted as a direct rejection marker, but rather as a supportive and rapidly assessable complementary biomarker candidate for infection-associated immune activity in clinically ambiguous situations where rejection and infection are difficult to distinguish. This interpretation may be made together with infection status, clinical findings, inflammatory markers, microbiological tests, imaging, and biopsy when rejection is clinically suspected.

In recent years, dd-cfDNA has gained increasing attention as a non-invasive biomarker for graft injury and early or subclinical rejection after solid organ transplantation [42]. Although dd-cfDNA is a promising marker in this context, it primarily reflects donor-derived graft cell injury rather than rejection alone. Therefore, dd-cfDNA is not completely rejection-specific; importantly for our study context, previous studies have reported that dd-cfDNA elevations may also occur in infectious conditions, as well as in other causes of graft injury such as ischemic injury and biliary or vascular complications [43,44,45]. This supports the concept that dd-cfDNA should be interpreted as a marker of graft injury rather than a rejection-exclusive biomarker.

In this context, anti-α-Gal IgM should not be considered an alternative to dd-cfDNA. Rather, it may represent a more accessible and lower-cost complementary biomarker candidate reflecting infection-associated humoral immune activity. In addition, IFNG and IL10 expression patterns may provide complementary information regarding immune activation. When interpreted together with dd-cfDNA, inflammatory markers, cultures, imaging, and clinical findings, these biomarkers may help contextualize whether graft injury is accompanied by an infection-associated or rejection-associated immune response.

The anti-α-Gal IgG antibody level was found to be higher in the group with liver disease before and after the transplantation compared to the healthy control group. Following the bacterial translocation observed in liver damage, the immune system’s encounter with these bacteria may cause an increase in anti-α-Gal IgG synthesis by plasma cells. Translocation of gram-negative bacteria is frequently observed in patients with liver cirrhosis [46]. It has been reported that the presence anti-α-Gal antibodies directed against bacterial α-Gal-containing structures was significantly associated with individuals with fibrosis stage III or greater, regardless of the etiology of their liver disease [47]. Patients with increased levels of anti-α-Gal antibodies have levels of endotoxin and other markers of bacterial exposure [48]. It is surprising that anti-α-Gal1,3 IgG levels were close to preoperative levels in both time periods after transplantation. The fact that these values do not vary in both infected and non-infected samples suggests the production of antibodies caused by a different stimulus other than bacteria such as the hepatitis virus.

Several limitations and future perspectives should be considered. It is necessary to plan studies that include a larger study population and take into account, as much as possible, the mechanism of action of the immunosuppressive treatment used. Since the present study was limited to liver transplant recipients, we could not determine whether the observed anti-α-Gal IgM dynamics are specific to liver transplantation. Future studies including other solid organ transplant cohorts, such as kidney and cardiac transplantation, and deceased donor liver transplantation cases may allow a more comprehensive evaluation of this issue.

Moreover, liver transplant recipients represent a highly complex and heterogeneous patient population, as they are often referred for transplantation due to severe and complicated underlying diseases and may be exposed to different pathogens before or during transplantation. Infectious complications in our cohort were also heterogeneous in terms of pathogen type, infection site, timing, recurrence, and clinical severity. Therefore, with currently available clinical information, it is not possible to comprehensively evaluate graft success or patient status in such a complex population based on only one or two parameters. For this reason, infection-related biomarker findings should be interpreted in the context of conventional clinical parameters, and both conventional and emerging biomarkers should be evaluated together in a complementary manner.

In conclusion, our findings suggest that longitudinal monitoring of IFN-g, IL-10, and anti-α-Gal IgM, when evaluated together with microbiological findings and conventional clinical parameters, may contribute to the assessment of immune activity, infectious complications, and post-transplant recovery in liver transplant recipients.

## Figures and Tables

**Table 1:** Demographic and Clinical Characteristics of Liver Transplant Patients.

Features of Patients	Quantity (%)
Gender	
Male	16 (62%)
Female	10 (38%)
Age	
Mean ± SD	50.7 ± 12.17
Median (Min–Max)	55 (23–67)
Type of Transplantation	
Living Donor	26 (100%)
Cadaver	0 (0%)
MELD Score	
Mean ± SD	17.73 ± 4.98
Median (Min–Max)	17 (8.10–28)
Diagnosis	
HBV	9 (34%)
HBV + HCC	4 (15%)
HCC	3 (12%)
Alcoholic Cirrhosis	3 (12%)
Cryptogenic Cirrhosis	3 (12%)
Autoimmune Hepatitis	2 (7.5%)
Budd–Chiari Syndrome	2 (7.5%)

SD: Standard Deviation; Min: Minimum; Max: Maximum; MELD: Model for End-stage Liver Disease.

**Table 2:** Duration of Tacrolimus and Everolimus Use of Patients in the 6-Month Postoperative Period.

Patient	1st Month	2nd Month	3rd Month	4th Month	5th Month	6th Month
1	T	T	T	T	T	T
2	T	T	T	T	T + E	T + E
3	T	T	T	T	T	T
4	T	T	T	T + E	T + E	T + E
5	T	T	T	T	T	T
6	T	T	T	T	T	T
7	T	T	T	T	T	T
8	T	T	T	T	T	T
9	T	T	T	T	T	T
10	T	T + E	T + E	T + E	T + E	T + E
11	T	T + E	T + E	T + E	T + E	T + E
12	T	T + E	T + E	T + E	T + E	T + E
13	T	T	T	T	T	T
14	T	T	T	T	T	T
15	T	T	T	T + E	T + E	T + E
16	T	T	T + E	T + E	T + E	T + E
17	T	T	T	T	T	T
18	T	T	T	T	T	T
19	T	T	T	T	T	T
20	T	T + E	T + E	T + E	T + E	T + E
21	T	T	T + E	T + E	T + E	T + E
22	T	T + E	T + E	T + E	T + E	T + E
23	T	T + E	T + E	T + E	T + E	T + E
24	T	T + E	T + E	T + E	T + E	T + E
25	T	T	T	T	T	T
26	T	T	T	T	T	T

T and E symbolize tacrolimus and everolimus, respectively. T + E represents periods in which both tacrolimus and everolimus were used.

**Table 3:** Features of Primers used in quantitative Real-Time PCR.

Gene	Reference Segment	Reference Position	Band Size (bp)
*IL-2*	NM_000586.3	489	85
*IL-4*	NM_000589.3	320	93
*IL-6*	NM_000600.3	816	98
*IL-10*	NM_000572.2	168	63
*TNF-a*	NM_000594.3	749	110
*IFN-γ*	NM_000619.2	1133	68
*FOXP3*	NM_014009.3	2191	89
*TREM1*	NM_018643.3	425	156
*CD14*	NM_000591.3	463	152
*HLA-G5*	NM_002127.5	680	384
*GAPDH*	NM_002046.5	842	130

**Table 4:** Biochemical parameters for patients at three different time points.

	Preop	Postop 1st Month	Postop 6th Month
ALT (U/L)	45.00 ± 31.73 *	70.19 ± 54.08 ^#^	31.81 ± 23.64
AST (U/L)	63.11 ± 48.56 ^$^	39.41 ± 26.89	27.63 ± 17.60
AFP (ng/mL)	9.56 ± 12.27 *^$^	1.94 ± 2.12	5.44 ± 15.02
PLT (K/uL)	104.93 ± 73.11 *^$^	232.74 ± 122.48 ^#^	169.85 ± 76.97
CRP (mg/dL)	1.17 ± 1.43 *	2.64 ± 2.93 ^#^	0.89 ± 1.54
Bilirubin D (mg/dL)	2.55 ± 2.47 *^$^	1.26 ± 1.06 ^#^	0.62 ± 0.72
Bilirubin T (mg/dL)	1.65 ± 1.60 *^$^	0.85 ± 0.83	0.81 ± 0.76

ALT, AST, AFP, PLT, CRP, Bilirubin D, and Bilirubin T data are presented as mean ± standard deviation. The ‘*’ symbol indicates the statistical differences between the Preop group and the Postop I group, the ‘^$^’ symbol indicates the statistical differences between the Preop group and the Postop VI group, and the ‘^#^’ symbol indicates the statistical differences between the Postop I group and the Postop VI group (*p* < 0.05).

**Table 5:** Timeline distribution of microorganisms detected in blood (B), respiratory tract (RT), urinary tract (UT), and surgical site (SS) samples of liver transplant patients.

	During Op.				Post-Op 1–3 Month				Post-Op 3–6 Month			
Pathogen	B	RT	UT	SS	B	RT	UT	SS	B	RT	UT	SS
Gram-positive bacteria												
*Streptococcus mitis*		1										
*Staphylococcus aureus*		1			1	1		1		1		
*Staphylococcus homini*					1							
*Staphylococcus* spp.		1		3								
Enterobacteriaceae												
*Escherichia coli*					1		1					
*Klebsiella pneumoniae*		1	2				2					
*Proteus mirabilis*								1				
Nonfermenter Gram-negative bacilli												
*Acinetobacter baumanii*					1				1			
*Pseudomonas* spp.											1	
*Pseudomonas aeriginosa*					1							
Fungus												
*Candida* spp.			2								1	
*Candida keyfr*											1	

**Table 6:** Average 2^-delta delta ct Values of Genes in Patients with and Without Rejection.

	R1 (Ort. ± SS)	NonR1 (Ort. ± SS)	R6 (Ort. ± SS)	NonR6 (Ort. ± SS)
IL -2	0.193 ± 0.07	0.262 ± 0.04	0.311 ± 0.11	0.387 ± 0.06
IL-4	0.329 ± 0.07	0.511 ± 0.10	0.282 ± 0.07	0.328 ± 0.03
IL-6	0.453 ± 0.15	0.718 ± 0.16	0.468 ± 0.14	0.617 ± 0.08
IL-10	1.559 ± 0.33	1.453 ± 0.18	1.252 ± 0.16	0.908 ± 0.09 ^#^
TNF-α	0.514 ± 0.06 *	0.792 ± 0.08	0.636 ± 0.09	0.745 ± 0.04
IFN-γ	0.214 ± 0.06	0.448 ± 0.11	0.384 ± 0.12	0.571 ± 0.08 ^#^
FOXP3	0.426 ± 0.14	0.488 ± 0.06	0.497 ± 0.10	0.458 ± 0.06
TREM1	0.714 ± 0.06	0.758 ± 0.05	0.601 ± 0.07	0.670 ± 0.06
CD14	0.908 ± 0.09	0.986 ± 0.05	0.837 ± 0.11	1.079 ± 0.10
HLA-G5	0.228 ± 0.102	0.154 ± 0.03	0.291 ± 0.11	0.266 ± 0.05

Shows mean ± standard error values in patients with and without rejection. R1: Mean gene expression levels of patients with rejection measured 1 month after surgery; NonR1: Mean gene expression levels of patients without rejection measured 1 month after surgery, R6: Mean gene expression levels of patients with rejection measured 6 months after surgery; NonR6: Mean gene expression levels of patients without rejection measured 6 months after surgery. * indicates the statistical significance of TNF-a gene between R1 and NonR1; # indicates the statistical difference of IL-10 and IFN-g genes between NonR1 and NonR6.

## Data Availability

The original contributions presented in this study are included in the article/Supplementary Material. Further inquiries can be directed to the corresponding author.

## Supplementary Materials

The following supporting information can be downloaded at: https://www.mdpi.com/article/10.3390/diagnostics16111635/s1, Table S1: Pathogen-specific descriptive anti-α-Gal IgM and IgG profiles in liver transplant recipients.
