# Supplementary material for: Monitoring Inflammatory Markers and Anti-α-Gal Antibodies in Liver Transplant Recipients: Implications for Infection and Rejection
Source: Diagnostics (Basel). 2026 May 27;16(11):1635. doi: 10.3390/diagnostics16111635 (PMC13256551; doi:10.3390/diagnostics16111635)
Supplement: Supplementary file 1 [file diagnostics-16-01635-s001.zip › diagnostics-4306205-supplementary.pdf]

Supplementary Table S1. Pathogen-specific descriptive anti-α-Gal IgM and IgG profiles in liver transplant recipients

Pathogen (patient n)	Pathogen detections	IgM preop mean	IgM 1M mean	IgM 6M mean	IgG preop mean	IgG 1M mean	IgG 6M mean
Klebsiella pneumoniae (n=4)	5	12,32	21,24	30,88	407,82	355,58	475,51
Escherichia coli (n=2)	2	58,09	32,06	17,47	489,23	606,41	622,82
Pseudomonas spp./P. aeruginosa (n=2)	2	91,35	54,55	27,26	442,31	373,08	390,77
Candida spp./C. kefyr (n=4)	4	91,05	66,76	65,5	428,65	376,99	367,63
Staphylococcus spp., Staphylococcus aureus, Staphylococcus hominis (n=4)	5	64,79	50,02	19,24	442,56	505,73	536,03
Staphylococcus aureus (n=4)	5	54,89	46,69	14,35	442,74	510,96	608,65
Acinetobacter baumannii (n=2)	2	52,75	52,1	53,95	299,87	459,74	622,82
Gram-positive bacilli (n=3)	3	49,86	63,38	10,53	590,26	453,08	485,9
Proteus mirabilis (n=1)	1	31,48	88,4	9,6	525,13	471,54	622,82
Streptococcus mitis (n=1)	1	91,7	92,5	81,75	549,74	540,26	622,82
Haemophilus influenza (n=1)	1	93,1	18,3	4,8	622,82	378,46	622,82
Cocci observed (n=1)	1	9,6	12,4	15,8	599,74	172,31	140,26
**Non-infection group (n=10)**	—	**62,78**	**66,02**	**16,28**	**302,01**	**329,46**	**281,41**

Note: Pathogen detections indicate the number of culture-positive microorganism detections/infectious episodes, not the number of patients. Patients with multiple microorganisms or recurrent infections may contribute to more than one pathogen category. Anti-α-Gal IgM and IgG values are presented as descriptive means at the predefined sampling time points. Formal pathogen-specific statistical comparisons were not performed because of the small and partially overlapping subgroups. The non-infection group includes patients without documented infectious complications.
